# Mortality risk in the population of HIV-positive individuals in Southern China: A cohort study

**DOI:** 10.1371/journal.pone.0210856

**Published:** 2019-02-11

**Authors:** Zhigang Zheng, Jinying Lin, ZhenZhen Lu, Jinming Su, Jianjun Li, Guangjie Tan, Chongxing Zhou, Wenkui Geng

**Affiliations:** 1 HIV/AIDS Program, Guangxi Zhuang Autonomous Region Center for Disease Prevention and Control, Nanning, China; 2 Guangxi Key Laboratory for the Prevention and Control of Viral Hepatitis, Guangxi Zhuang Autonomous Region Center for Disease Prevention and Control, Nanning, China; 3 Guangxi People’s Hospital, Nanning, China; 4 Guangxi Health and Family Planning Committee, Nanning, China; Stellenbosch University, Desmond Tutu TB Centre, SOUTH AFRICA

## Abstract

To evaluate the mortality risk in the HIV-positive population, we conducted an observational cohort study involving routine data collection of HIV-positive patients who presented at HIV clinics and multiple treatment centers throughout Guangxi province, Southern China in 2011. The patients were screened for tuberculosis (TB) and tested for hepatitis B (HBV) and C (HCV) virus infections yearly. Following the registration, the cohort was followed up for a 60-month period till the end-point (December 31, 2015). Univariable and multivariable Cox proportional hazards regression models were used to analyze the hazard ratio (HR) and 95% confidence interval (95% CI) for mortality after adjusting for confounding factors stratified by patients’ sociodemographic and behavioral characteristics. HRs were compared within risk-factor levels. With the median follow-up of 3.7-person years for each individual, 5,398 (37.8%) (of 14,293 patients with HIV/AIDS) died; among whom, 78.4% were antiretroviral therapy (ART)-naïve; 43.6% presented late; and 12.2% and 3.3% of patients had *Mycobacterium tuberculosis* (MTB) and HBV and HCV co-infection, respectively. Of individuals with CD4 counts, those with CD4 count >350 cells/μL formed 14.0% of those who died. Furthermore, gender [multivariable HR (95% CI):1.94 (1.68–2.25)], Han ethnicity [2.15 (1.07–4.32)], illiteracy [3.28 (1.96–5.5)], elementary education [2.91 (1.8–4.72)], late presentation [2.89 (2.46–3.39)], and MTB co-infection [1.28 (1.10–1.49)] strongly increased the all-cause mortality risk of HIV-positive individuals. The HR for ART-based stratification was 0.08 (0.07–0.09); and for HBV and HCV co-infection, HR was 1.02 (0.86–1.21). The findings emphasized that accessibility to HIV testing among high-risk populations and screening for viral hepatitis and TB co-infection are important for the survival of HIV-positive individuals. Initiating early ART, even for individuals with higher CD4 counts, is advisable to help increase the prolongation of lives within the community.

## Introduction

With the emergence of the human immunodeficiency virus (HIV) pandemic in the 1980s, a major upsurge in tuberculosis (TB) cases and TB-related mortality has been observed in many countries [1]. TB is the most common opportunistic infectious disease among people living with HIV in developing countries [2]. *Mycobacterium tuberculosis* (MTB) infection is the leading cause of death among HIV-positive individuals [3]. Among patients with TB and HIV co-infection in some countries, more than 50% have died during the process of anti-TB therapy, the death mainly occurred within two months of TB diagnosis [4–6]. Although ART has been proved to be a crucial intervention to reduce the risk of death among HIV-positive TB patients [4,7], in some resource-limited countries with ART coverage less than 30%, heavy disease burden caused by the higher mortality of HIV-positive patients with TB have resulted [8–12]. In cases involving TB co-infection with HIV, ART can further decrease treatment adherence of anti-TB drugs [13], thus increasing the risk of death, and persistent transmission among these patients [14,15].

In China, one of 41 countries with the highest HIV and TB co-infection (HIV/TB) burden, the World Health Organization (WHO) estimated that the proportion of HIV-positive TB patients who initiated ART was 85% in 2016 [16]. Guangxi is a province in Southern China with simultaneously high HIV prevalence and a TB pandemic, where currently more than 110,000 people were registered with HIV, while more than 50,000 cases have been registered as active TB patients in the National Legal Mandatory Report System in 2016. The disease burden caused by HIV/TB in Guangxi is ranked number one among the 31 provinces in China [17]. Furthermore, Guangxi is also a place with high incidence of viral liver disease and hepatocellular carcinoma [18]. In addition, compared with the 35.5% of patients with late HIV presentation in China, the percentage with late presentation was more than 51% of the total registrations across Guangxi from 2010–2014 [19], ranking Guangxi as number two among the 31 provinces in China. Those who present later have a higher risk of TB co-infection and a higher risk of mortality as well. Although we know that more than 30% of those with HIV died of MTB co-infection worldwide, little is known about the quantitative mortality risk in the population of those with HIV/TB, or hepatitis disease co-infection, or late presentation Therefore, the aim of this paper was to evaluate the mortality risk of HIV-positive individuals. Our findings will provide a different academic approach to estimating the risk of mortality among HIV-positive individuals, and add to the literature on mortality risk associated with HIV in a region with high HIV, TB, and hepatitis prevalence, as well as with frequency of late presentation.

## Materials and methods

### Participants

**Fig 1:**
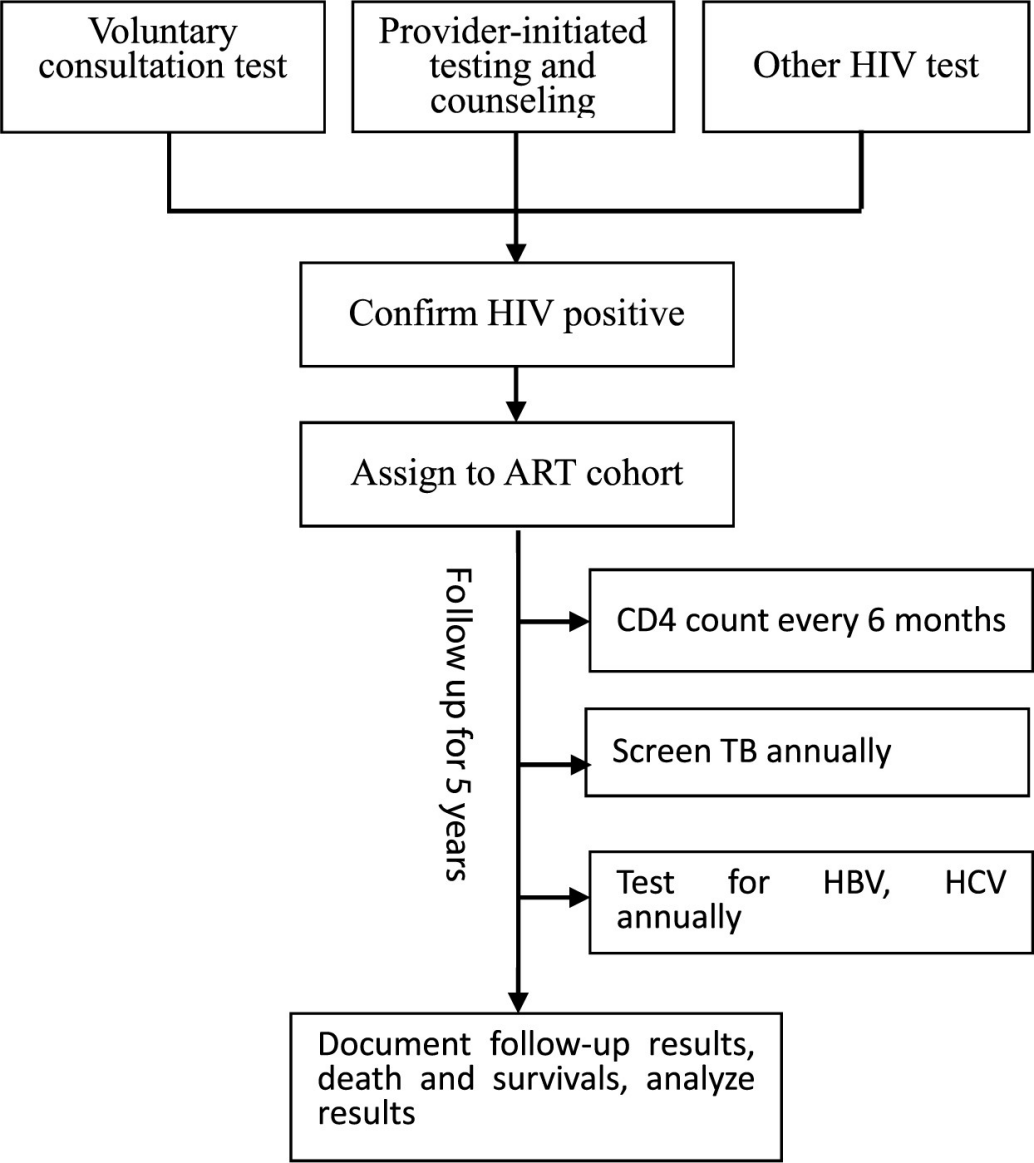
Study profile diagram. This diagram shows the flow of HIV/AIDS being assigned to antiretroviral therapy (ART), followed-up, and the main bio-indicators have been analyzed.

Individuals who have had HIV high-risk behaviors underwent a voluntary consultation test (VCT) at local HIV clinics or Center for Disease Control and Prevention (CDC) system. Patients who have had provider-initiated testing and counseling in hospitals (PITC) in Guangxi were sampled for the first HIV blood test, and the confirmation of HIV infection was made by the second blood test by western blot if the first sample tested positive. Individuals were registered with a treatment cohort if they were confirmed HIV positive. Participants in this study were started on a standard treatment regimen between January 1, 2011 and December 31, 2011; TB diagnosis information was then collected annually. Participants inclusion criteria were: 1. Being ≥15 years old; 2. Having completed a new registration; 3. TB status being available yearly; and 4. HBV and HCV test results were available yearly. Clinical review of patients was required every six months; we included follow-up data up to December 31, 2015 (Fig 1. Study profile diagram).

### TB and hepatitis co-infection

Sputum and blood were sampled for TB, HBV, and HCV testing. For the diagnosis of TB co-infection, chest-X ray, sputum smear examination, and microscopy were used; however, some samples were cultured for MTB in larger, centrally-located laboratory facilities when smears were negative. TB co-infection was confirmed when acid-fast staining was positive or MTB was found in the culture. HBV and HCV were tested for using enzyme-linked immunosorbent assay (ELISA), a commercial third generation, rapid, qualitative, two-site sandwich immunoassay test device was employed according to the manufacturer's instructions (Tulip Diagnostics (P), Ltd., Goa, India). The kit was used to detect hepatitis B surface antigen (HBsAg) to confirm HBV and to test for antibodies specific against HCV infection in serum or plasma by using a multiepitope recombinant peptide antigen that is broadly cross-reactive to all major HBV and HCV genotypes. In each county, there is a treatment center where HIV care is provided for HIV positive individuals, facility for smear microscopy is also provided for the diagnosis of TB, while HBsAg and antibody test for HCV are also validated in the laboratory.

### Data collection

Data were collected from the HIV observation cohort that was registered for HIV/AIDS in 2011, in Guangxi. Standard follow-up information recorded by program staff and reported to the system included identification card (ID) number, death, ART, time of follow-up, CD4 cell count, TB and hepatitis status, and WHO clinical disease stage. Household information such as demographic, socio-economic status, and health data were collected at both the baseline and follow-ups; blood samples were collected yearly to test for hepatitis. Laboratory testing of blood samples was performed according to the national HIV laboratory testing operational instructions. Person year (PY) was used to measure the follow-up time from baseline to set point for each individual. If the time period of followed-up was one year from baseline to set point for one individual, we defined the individual was followed-up by one person year.

HIV staff in county CDCs and local clinical sites completed the baseline data collection, then entered to a system and sent to Guangxi CDC through an Internet-based electronic data collection system,

If participant was lost to follow-up (3 months absence from a clinic since last visit), a site visit was conducted by clinic staff to the participant’s address to confirm their absence or death. Information collected during the site visit included the date of the last visit to the clinic or date of death.

The data was managed by Guangxi CDC, we have full right to get access to the data.

### Ethics statement

We de-identified the dataset and analyzed anonymously. The informed consent that included information on sampling to test for HIV, CD4 count and their information utilization was signed by individuals in the process of baseline data collection. If the individuals were less than 18 years, their parents signed the informed consents.

This study was approved by the Committee of Ethics of the Guangxi Zhuang Autonomous Region Center for Disease Control and Prevention in Nanning.

### Statistical analysis

We used the stepwise selection method to choose the variables to include in the multivariable model. The data was stratified by gender, age group, ART, education, late presentation, TB, and HBV and HCV co-infection. Person-year of follow-up and mortality associated with HIV were calculated by stratifications.

We calculated the regression coefficient to identify the predictors of mortality; estimated the hazard ratio (HR, refers to the mortality risk for the individuals at time t+1 which was survived at time t) for mortality using univariable and multivariable Cox analysis, and determined the risk estimation with the calculation of HR values and 95% confidence interval (CI) for each stratification to understand the degree of the risk of mortality among HIV positive individuals. We tested for the goodness-of-fit of the whole regression model, the effect of which was good (χ^2^ = 1999.02, *P*<0.0001). Data were analyzed by using R software (version 3.2.2, R Foundation for Statistical Computing, Vienna, Austria) and SPSS 22.0 (IBM Inc. Armonk, NY, USA), significance level was set at 0.05, and all hypothesis testing were two-sided.

## Results

### Demographic and follow-up information

In total, 14,293 patients were recruited into the current study, of whom 10,128 (70.9%) were male; 40,362.0 PYs were recorded over the 60-month follow-up period (median [M]: 3.71, inter-quartile range [IQR]: 1.08, 4.19). The mean CD4 count was 348.4 [standard deviation (SD): 248.7], ART coverage was 56.7% (8102/14293). The 25–34 years age group comprised the majority (21.2%) for late presentations, the number of patients with available CD4 count data was 60.5% (6245/10318); 4.8% (685/14293) and 2.0% (280/14293) were co-infected with HBV and HCV, respectively. Overall, 9.7% of patients were MTB positive; among the HIV/TB co-infected patients, 8% (1150/14293) and 1.7% (244/14293) were co-infected with pulmonary TB (PTB) and extra PTB (Ext PTB), respectively. Table 1 shows the demographic information for the HIV/AIDS observation cohort.

**Table 1:** Demographic information for the HIV/AIDS observation cohort in Guangxi.

Items	All patients	HIV	AIDS
Total case (%)	14,293(100.0)	6,696(46.8)	7597(53.2)
Of which died(%)	5398(37.8)	2460(36.7)	2938(38.7)
Median of PY of follow-up	3.71	3.81	3.54
IQR	1.08, 4.19	1.34, 4.22	0.96, 4.16
Age(Year)			
15–24 Year(%)	797(5.6)	557(8.3)	240(3.2)
25–34 Year(%)	3024(21.2)	1558(23.3)	1466(19.3)
35–44 Year(%)	2815(19.7)	1214(18.1)	1601(21.1)
45–54 Year(%)	2171(15.2)	894(13.4)	1277(16.8)
55–64 Year(%)	2640(18.5)	1098(16.4)	1542(20.3)
65- Year(%)	2846(19.9)	1375(20.5)	1471(19.4)
Gender			
Male (%)	10128(70.9)	4490(67.1)	5638(74.2)
Female(%)	4165(29.1)	2206(32.9)	1959(25.8)
Ethnics			
Han(%)	9312(65.2)	4369(65.2)	4943(65.1)
Zhuang(%)	4333(30.3)	1989(29.7)	2344(30.9)
Yao(%)	368(2.6)	198(3.0)	170(2.2)
Others(%)	280(1.9)	140(2.1)	140(1.8)
Education			
Illiterate(%)	929(6.5)	481(7.2)	448(5.9)
Elementary School(%)	6193(43.3)	2885(43.1)	3308(43.5)
Junior High School(%)	5596(39.2)	2577(38.5)	3019(39.7)
High School(%)	1220(8.5)	575(8.6)	645(8.5)
Community College and Above(%)	355(2.5)	178(2.7)	177(2.3)
CD4 count mean (SD) (cell/μL)	348.4(248.7)	508.1(227.2)	245.3(203.4)
Late presentation			
Late(%)	6245(43.7)	80(1.2)	6165(81.2)
Not late(%)	4073(28.5)	3852(57.5)	221(2.9)
Missing(%)	3975(27.8)	2764(41.3)	1211(15.9)
ART			
Yes(%)	8102(56.7)	2803(41.9)	5299(69.8)
Naïve(%)	6191(43.3)	3893(58.1)	2298(30.2)
Hep co-infection			
Hep B(%)	685(4.8)	239(3.6)	446(5.9)
Hep C(%)	280(2.0)	97(1.4)	183(2.4)
MTB co-infection			
PTB	1150(8.0)	242(3.6)	908(12.0)
Ext-PTB	244(1.7)	97(1.4)	147(1.9)

Abbreviation: IQR, inter quartile range; ART, antiretroviral therapy; Hep, hepatitis; MTB, mycobacterium tuberculosis; PTB, pulmonary tuberculosis; Ext-PTB, extra pulmonary tuberculosis.

### Mortality and risk factors

In total, 5,398 HIV positive individuals died within 5-years of follow-up in this cohort, and mortality was 13.37/100 (5398/40362.2) PYs. Among these, 78.4% were ART naïve, 43.6% died due to late presentation; MTB as well as HBV and HCV co-infection caused 12.2% and 3.3% of deaths, respectively. Based on the empirical experience, social demographic variables such as gender, ethnic group, education, and behavioral characteristics such as late presentation, MTB co-infection, ART as well as HBV and HCV co-infection were included in the calculation of the regression coefficient (β) to identify predictors of mortality. Gender (β = 0.696, *P*<0.0001), ethnic group (β = 0.072, *P* = 0.01), education (β = 0.434, *P*<0.0001), late presentation (β = 1.065, *P*<0.0001), and MTB co-infection (β = 0.245, *P* = 0.001) have been identified as risk factors of mortality among HIV positive individuals; and ART (β = -2.462, *P*<0.0001) has been identified as a protective factor against mortality among HIV-positive individuals. However, hepatitis was not associated with mortality among HIV-positive patients in this cohort (β = 0.007, *P* = 0.94) (Table 2).

**Table 2:** Multivariable analysis of risk factors of mortality among HIV/AIDS individual in the 5-year follow-up.

Factors	β	SE	Wald	df	*P* value	Exp(β)	95.0% CI for Exp (β)	
							Lower limit	Upper limit
Gender	0.696	0.07	89.46	1	<0.0001	0.50	0.43	0.58
Ethnic	0.072	0.03	6.55	1	0.01	0.93	0.88	0.98
Education	0.434	0.04	130.56	1	<0.0001	0.65	0.60	0.70
Late presentation	1.065	0.08	172.95	1	<0.0001	0.34	0.29	0.40
ART	-2.462	0.08	1031.00	1	<0.0001	11.73	10.09	13.63
Hep co-infection	0.007	0.09	0.01	1	0.94	1.01	0.85	1.19
MTB co-infection	0.245	0.08	10.39	1	0.001	0.78	0.67	0.91

Abbreviation: ART, antiretroviral therapy; Hep, hepatitis; MTB, mycobacterium tuberculosis.

### Population risk of mortality

**Fig 2:**
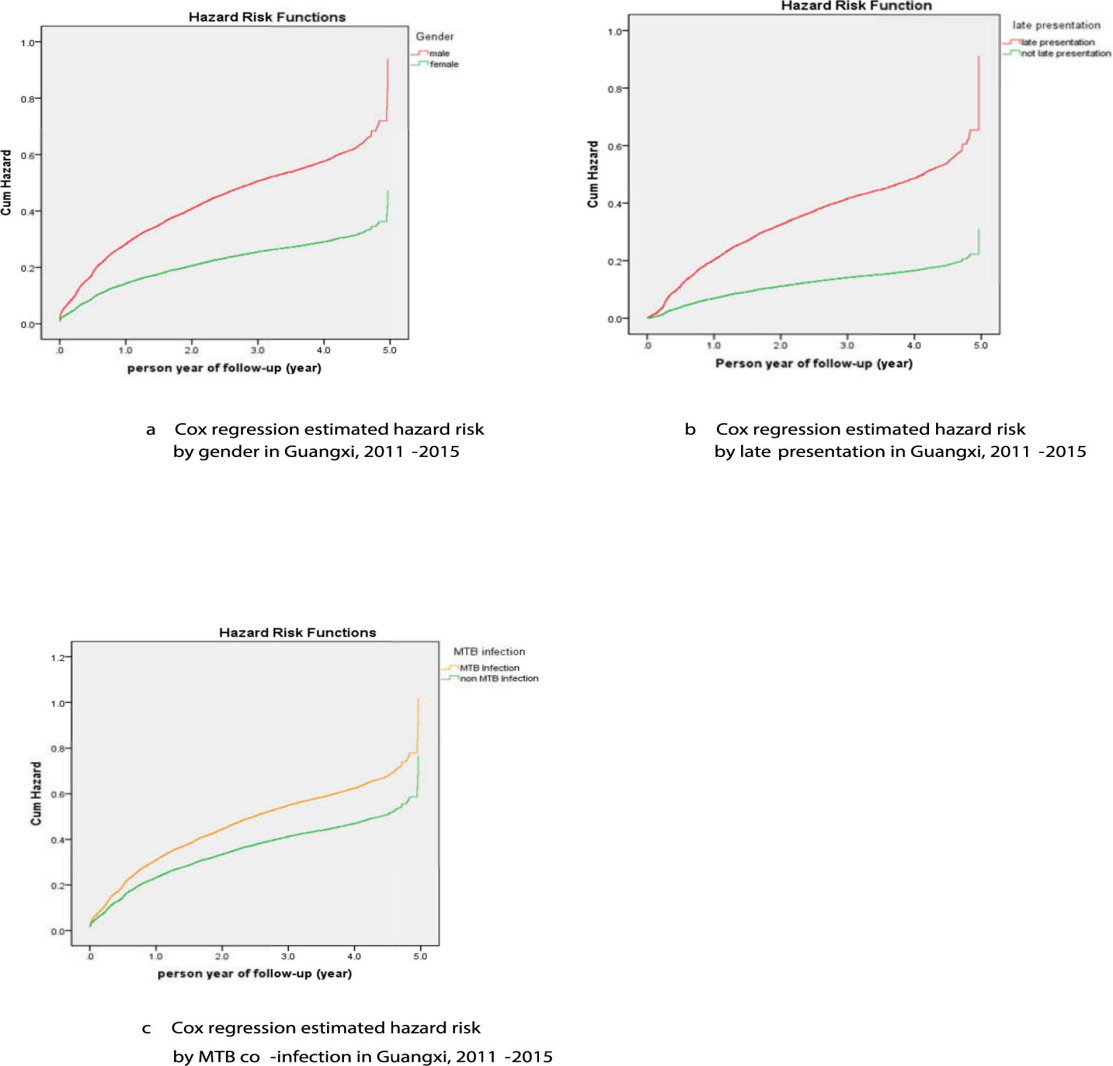
Cox regression estimated hazard risk by different social demography and behavior characteristics in Guangxi, 2011–2015. Those figures shows different mortality risk in different social demography and behavior characteristics groups such as gender (Fig 2A), HIV late presentation (Fig 2B), mycobacterium tuberculosis (MTB) coinfection (Fig 2C), on ART (Fig 2D), ethnic (Fig 2E), education (Fig 2F), and hepatitis coinfection (Fig 2G).

We calculated within stratification HR values of mortality using univariable and multivariable Cox regression models; hazard risk of mortality in males was 1.98 times higher than in females (95% CI: 1.85,2.12) (*P*<0.0001) (Fig 2A), the result of univariable analysis was consistent with the multivariable analysis. The HR value of mortality in late presentation, stratified by MTB co-infection, was 2.9, and 1.32 times according to univariable analysis compared with those who presented on time and those without MTB co-infection. We also found coherence with the multivariable analysis results (Fig 2B and 2C). ART was a protective factor against mortality, and the mortality risk of individuals on ART was 0.12 (95% CI: 0.11, 0.13) (*P*<0.0001) times compared to that of the ART-naïve individuals in univariable analysis (Fig 2D). Compared to other ethnic groups, the HR value among the Han was 1.51 (95% CI: 1.20, 1.90) times, which was significantly different (*P*<0.0001) at univariable analysis, and was consistently concordant with that of the multivariable analysis results (Fig 2E); similar mortality risk results was observed in those who are illiterate, and those with elementary education, as compared to the Community College and above education participants (Fig 2F); the *P* value among the individuals co-infected with hepatitis was not consistent between univariable and multivariable analysis (Table 3), co-infected with hepatitis was considered as a confounding factor of causing mortality (Fig 2G).

**Table 3:** Univariable and multivariable Cox regression analysis of mortality among HIV observation cohort.

	univariable analysis in all-cause death			multivariable analysis in all-cause death		
Characteristics	βvalue	HR value (95%CI)	*P* value	βvalue	HR value (95%CI)	*P* value
Gender						
Male	0.69	1.98 (1.85,2.12)	< 0.0001	0.66	1.94 (1.68,2.25)	< 0.0001
Female	—	1	—	—	1	—
Ethnics						
Han	0.41	1.51 (1.20,1.90)	< 0.0001	0.77	2.15 (1.07,4.32)	0.03
Zhuang	0.27	1.31 (1.04,1.65)	0.02	0.69	1.99 (0.99,4.02)	0.06
Yao	0.33	1.39 (1.05,1.85)	0.02	0.39	1.48 (0.65,3.38)	0.36
Others	—	1	—	—	1	—
Education						
Illiterate	1.48	4.39 (3.37,5.74)	< 0.0001	1.19	3.28 (1.96,5.5)	< 0.0001
Elementary school	1.21	3.35 (2.59,4.33)	< 0.0001	1.07	2.91(1.8,4.72)	< 0.0001
Junior High school	0.58	1.79 (1.38,2.31)	< 0.0001	0.4	1.49 (0.92,2.42)	0.11
High school	0.44	1.55 (1.18,2.05)	0.002	0.22	1.25 (0.74,2.13)	0.41
Community college and above	—	1	—	—	1	—
Late presentation						
Late	1.06	2.90 (2.66,3.16)	< 0.0001	1.06	2.89 (2.46,3.39)	< 0.0001
Not late	—	1	—	—	1	—
ART						
Yes	-2.13	0.12 (0.11,0.13)	< 0.0001	-2.51	0.08 (0.07,0.09)	< 0.0001
Naïve	—	1	—	—	1	—
Hep co-infection						
Yes	0.02	0.98 (0.84,1.14)	0.77	0.02	1.02 (0.86,1.21)	0.82
Naïve	—	1	—	—	1	—
MTB co-infection						
Yes	0.28	1.32 (1.22,1.44)	< 0.0001	0.25	1.28 (1.10,1.49)	0.001
Naïve	—	1	—	—	1	—

Abbreviation: ART, antiretroviral therapy; Hep, hepatitis; MTB, mycobacterium tuberculosis.

## Discussion

In this observational cohort of over 14,000 individuals, we identified demographic predictors such as gender, Han ethnicity, illiteracy, elementary education, late presentation, and MTB co-infection, which were independent risk factors of all-cause mortality among HIV positive individuals during the 5-year follow-up. The HRs of mortality were higher among males, Han ethnicity, low education, and late presentation, but lower with higher ART coverage. Hepatitis co-infection was not associated with an increase in mortality.

The mortality rate in this cohort was similar to that of a contemporary cohort in China [20]. The high mortality was due to the high percentage of MTB (including PTB and Ext-PTB) co-infection, late presentation, and insufficient ART coverage. In our cohort, although a higher percentage of MTB co-infected patients had initiated ART (63.4% in MTB co-infection vs. 56.0% in mono-HIV positive patients), higher mortality rate was observed in this population (17.6/100 PYs in MTB co-infection vs. 12.9/100 PYs in mono-HIV positive patients). Based on the multidrug-resistant TB (MDR-TB) surveillance results in 2016, the percentage of MDR-TB in Guangxi was 7.7% (153/1992), which was higher than the prevalence of MDR-TB in China (7.7% vs. 5.7%) [21]. Further research should be focused on TB treatment coverage and the role of MDR-TB among the HIV/TB co-infected cohort, to understand their contribution to HIV mortality.

Although 33% of HIV positive patients had died of MTB co-infection [16], the accuracy of the hazard risk was less certain. In this cohort, we found a significantly higher in hazard risk of mortality among those co-infected with MTB, which has provided an accurate estimation of mortality risk for HIV positive individuals with TB co-infection. An estimated 11% of incident TB cases in 2015 were HIV positive worldwide and notified TB cases as a percentage of those newly enrolled in HIV care was 2.7% in China [16]. However, prevalence analysis showed that HIV infection among those with PTB was 3.3% [22], while the prevalence of TB in HIV positive individuals was 7.2% [23]. In this cohort, a more serious TB prevalence of 9.75% (1394/14293) among HIV positive individuals was found in Guangxi, which was significantly higher than the results of a previous meta-analysis in Guangxi province [24]. The higher percentage in this study might be because of the imputation methods used to address missing and inconsistent MTB co-infection data, which might have influenced the outcomes. The findings urge for the development of a comprehensive HIV/TB co-infection control strategy, and more practical collaborative HIV/TB activities in such a high TB and HIV prevalence setting.

Low CD4 count or late presentation always leads to low ART uptake and late HIV care, resulting in serious damage to the immune system in HIV/AIDS [25], this is inevitably linked to higher mortality [26,27]. The proportion of late presentation of HIV positive patients in Guangxi ranked second among the 31 provinces in China, it was much higher than the percentage of late presentation in Portugal [28], Florida (in the United States) [29], and Brazil [30]. Beside the high HR of mortality in late presentation, the proportion of late HIV presentation accounted for 78.6% (2353/2995) of all-cause mortality among HIV positive individuals who had CD4 count data in Guangxi, these results are comparable to findings in Brazil in 2011 [30].

Many publications have proven that ART is an important intervention tool to reduce mortality among HIV positive individuals [31–33], our finding was also consistent with the results of previous studies. Since 2016, Guangxi has initiated the “Treat for All” HIV treatment strategy: HIV positive individuals will receive ART regardless of their CD4 count. Based on surveillance results, ART coverage was 77% in 2016 in Guangxi; ART coverage was lower in this cohort under the algorithm of “Treat for All” strategy. The primary criterion for ART initiation from 2012–2015 was a CD4 count of ≤350 cells/μL. Individuals with CD4 count >350 cells/μL were not eligible for ART, and they accounted for 11.1% of the ART naïve individuals at the time; while individuals with CD4 counts >350 cells/μL accounted for 14.0% of those who had CD4 count data available, and who had died. Among the patients with CD4 count data available, 47.5% (1821/3837) of patients who did not initiate ART had CD4 counts <200 cell/μL, and they contributed 43.3% of mortality in this cohort.

Like other researches from Brazil, Italy, and Vietnam [34–37], we found that HIV positive males had a higher hazard of mortality, and this familiar finding may relate to how males access healthcare at a later stage, have more frequent co-infections, and more acquired immunodeficiency syndrome (AIDS)-related diseases, and a higher potential of lost to follow-up than in females [38].

After adjusting for the confounding factors, only those of Han ethnicity, stratified by illiteracy and elementary school, had a higher risk of mortality in the Cox multivariable model. The reason the Han ethnic group had a higher mortality risk may relate to their different customs, including HIV care-seeking behaviors, among others. People with lower educational levels were usually not aware of the importance of condom use, the HIV status of their sexual partners, the importance of the adherence of ART for life, or have reduced access to treatment clinics due to their remote location and the associated lengthy commute, and social stigma, that would be associated with higher risk of mortality.

The association of HBV and HCV co-infection with all-cause mortality in HIV positive individuals has been reported in previous studies [39], but they have shown that all-cause mortality was only higher in triple infection (co-infected with HIV, HBV, and HCV simultaneously), and those with HCV co-infection, but not in those with HBV co-infection [40]. The inconsistent finding in our study suggest that more details should be extracted from the data, and that the risk of mortality should be stratified by HBV and HCV co-infection, respectively to understand more about the impact of hepatitis co-infection.

There were also some limitations to our study. First, although potential confounding factors were adjusted for with Cox multivariable method, there were still some unknown and unassessed confounders, which could be generating bias. Second, refined definitions of co-infection status might not be clear for clinic physicians; even with the sample size of more than 6,900 individuals, there were still 51% of individual who were missing test results for hepatitis, which could affect the outcome potentially.

## Conclusion

The important strength of our study was the determination of mortality, the risk factors, and the quantitative mortality of risk among different stratifications of behavior, and demographic characteristics. A significantly higher mortality was observed among HIV positive individuals with TB co-infection (HR = 1.28), late presentation (HR = 2.89), while ART was a protective factor against mortality (HR = 0.08). These findings emphasize the importance of improving HIV test accessibility for high risk populations as early as possible, intensifying TB and hepatitis case detection among HIV positive patients sooner, initiating ART even at higher CD4 count, to save more lives in the community.

## Supporting information

S1 File 2011 HIV positive individuals2. (XLS)
